# Study of Antidepressant and Sedative-Hypnotic Activity of Hydroalcoholic Extract of *Asperugo* *procumbens* L. Aerial Parts in Mice

**Published:** 2013

**Authors:** Seyedeh-Atefeh Mirshafa, Mohammad Azadbakht, Nematollah Ahangar

**Affiliations:** a *Pharmaceutical Sciences Research Center and Department of Toxicology and Pharmacology, Faculty of Pharmacy, Mazandaran University of Medical Sciences, Sari, Iran. *; b *Department of Pharmacognosy, Faculty of Pharmacy, Mazandaran University of Medical Sciences, Sari, Iran. *

**Keywords:** Depression, *Asperugo procumbens *L., Forced swimming test, Tail suspension

## Abstract

*Asperugo procumbents *L. has been used in Iranian traditional medicine for the refreshing, tranquillizing and mood elevating activities. The present study was undertaken to evaluate the antidepressant and sedative-hypnotic potential of acute administration of the hydroalcoholic extract of this plant in mice. Additionally, the effects of flumazenil on the hypnotic activity of the extracts were evaluated. None of the doses of the extract could significantly reduce immobility time in comparison with control group in antidepressant tests. In hypnotic test, 250 and 400 mg/kg dose ssign ificantly increased pentobarbital-induced sleeping time compared to vehicle. All of the doses of the extract significantly reduced the latency to sleep in comparison to the vehicle. Flumazenil reversed the augmented effects of extracts in pentobarbital-induced hypnotic test. The results of the present study indicate the low antidepressant and good sedative-hypnotic effects of the hydroalcoholic extract of *Asperugo procumbens *aerial parts in mice and that the central benzodiazepine receptors are involved in the sedative-hypnotic effects of this plant.

## Introduction

Depression is an important health care problem in the world that is characterized by several signs such as intense sadness, despair, and recurrent thoughts of death or suicide. Prevalence of this disorder is about 13-20% of the population (1). Approximately two third of depressed patients has suicide thoughts and 10- 15% of whom attempt suicide before the age of 40 (2). Although several synthetic drugs are available for treatment of depression, side effects such as dry mouth, hypotension, fatigue, sexual dysfunction and drowsiness limit the use of these treatments (3). In addition, the success rate of medication is low and at least 40% of the patients do not respond to the antidepressant drugs (4). Therefore, researches for new antidepressant drugs with fewer side effects are needed. 

Insomnia, defined as persistent difficulty in falling or staying asleep that affects daytime function, can induce significant psychological and physical disorders. Most patients engage in long-term use of benzodiazepines analogs to treat insomnia. But these drugs have limited benefits with obvious side-effects, such as impaired cognitive function, memory and general daytime performance (5). In addition, long-term administration results in tolerance and dependence. 

Numerous herbal medicines are recognized as active in the central nervous system (CNS) and they have at least a hypothetical potential to affect chronic conditions such as anxiety, depression, headaches or epilepsy, that do not respond well to conventional treatments (6, 7).

People from different regions of the world have used herbal medicines to alleviate affective disorders for many years and as a consequence, the search for novel pharmacotherapy from medicinal plants has progressed significantly in the past decade (8). An increasing number of herbal productshave been introduced into psychiatric practice.

*Asperugo procumbens *L. (Boraginaceae) more commonly known as German-madwort, is a herb with a slender stem that can grow up to 90 cm in length. The aerial parts of this herb are being used in Iran as a traditional medicine for treatment of skin infections, to strengthen the nervous system, refreshing, tranquillizing and mood elevating activities, as well as antispasmodic (9, 10). There are, however, no published reports regarding the antidepressant and tranquilizing activities of the plant. The aim of this study was to evaluate the antidepressant and sedative-hypnotic potential of acute administration of the *Asperugo procumbens *L. hydroalcoholic extract (AHE) in forced swimming test (FST), tail suspension test (TST) and pentobarbital-induced hypnotic test in mice.

## Experimental

*Preparation of the plant material*

Aerial parts of *A. procumbens *were collected from a grocery in Sari, Mazandaran.The plant was identified and authenticated by Dr. M. Azadbakht from the Department of Pharmacognosy, Faculty of Pharmacy, Mazandaran University of Medical Sciences (Sari, Iran). For preparation of hydroalcoholic extract, air-dried and powdered aerial parts of the plant (50 g) were macerated with adequate ethanol 70º for 72 h. Then extraction was done by the perculation method. The extract was then evaporated in a rotating evaporator and freeze dryer to give a residue (3 g). The residue was dissolved in normal saline: tween 80 (9: 1) for final suitable concentrations.

*Drugs*

Imipramine, diazepam, flumazenil and pentobarbital were purchased from Sigma Company (St Louis, MO, USA). Imipramine and pentobarbital were dissolved in saline: tween 80 (9:1). Diazepam and flumazenil were suspended in the solvent. All the solutions were freshly made on the day of testing and administered to a final volume of 10 mL/kg body weight of mice.

*Animals*

Male Swiss Albino mice (Institute Pasteur, Amol, Iran) weighing 20-30 g were housed at room temperature in a 12 h light/dark cycle. Food and water were available *ad libitum. *Tests were performed only after the mice had acclimated to the above environment. Number of animals in each group was 5-6. All experiments were conducted between 8:00 and 13:00 every day to avoid any temporal factor. Each animal was used for only one experimental condition. All experiments were carried out in accordance with international guideline outlined in the Guide for the Care and Use of Laboratory Animals (11).

*Forced swimming test*

The FST is the most widely used pharmacological model for assessing antidepressant activity. This method is based on the observation of animals exposed to a situation of forced swimming, in which they become passive and immobile after a period of vigorous activity (struggling), producing only the movements required to keep their heads above the water. The FST was carried out on mice according to the method of Porsolt *et al*. (12, 13). Swimming sessions were conducted by placing the animals in individual Plexiglass cylinders (40 cm high, 20 cm diameter) containing 20 cm of water at 24 ± 1 ºC. All animals were forced to swim for 6 min, and the time spent in immobility during the last 5 min of a 6 min observation period was recorded manually by the competent observer. The animals were treated with the extracts (25, 50, 250 and 400 mg/kg, IP), imipramine (15 mg/kg, IP) or vehicle, 45 min before the test. 

*Tail suspension test*

The total duration of immobility induced by tail suspension was measured according to the method described by Steruet *et al*. (14). Briefly, mice both acoustically and visually isolated were suspended 58 cm above the floor by adhesive tape placed approximately 1-2 cm from the tip of the tail. Immobility time was manually recorded during a 5 min period (15). Mice were considered immobile only when they hung passively or stayed completely motionless. Conventional antidepressants decrease the immobility time in this test. The animals were treated with the extracts (25, 50, 250 and 400 mg/kg, IP), imipramine (15 mg/kg, IP) or vehicle, 45 min before the test.

*Pentobarbital-induced hypnotic test*

Righting reflex is a useful measure for assessing whether or not animals are asleep. Mice were given a single IP dose of the vehicle, diazepam (2 mg/kg) as the reference drug or different concentrations of the extract (50, 250, 400 mg/kg).These treatments were carried out 30 min before challenging the animal with IP injection of pentobarbital (40 mg/kg). The latency of the loss of the righting reflex and the total sleeping time (the time between the loss and the recovery of the righting reflex) were determined for each mouse. The mouse was considered as being awake if it could right itself (return to upright position). Once a mouse righted itself, it was placed on its back once more and allowed to right a second time for confirmation (16).

To investigate the possible mechanism involved in the hypnotic activity of *Asperugo procumbens *L. hydroalcoholic extract (AHE), the animals were pretreated with flumazenil (10 mg/kg), antagonist of GABA_A_–benzodiazepine receptor*.*

*Statistics*

All the data were expressed as mean+SEM. Comparison between groups wereperformed by one-way ANOVA followed by Tukey’s HSD test. P-values of less than 0.05 implied significance.

## Results

*Forced swimming test*

**Figure 1:**
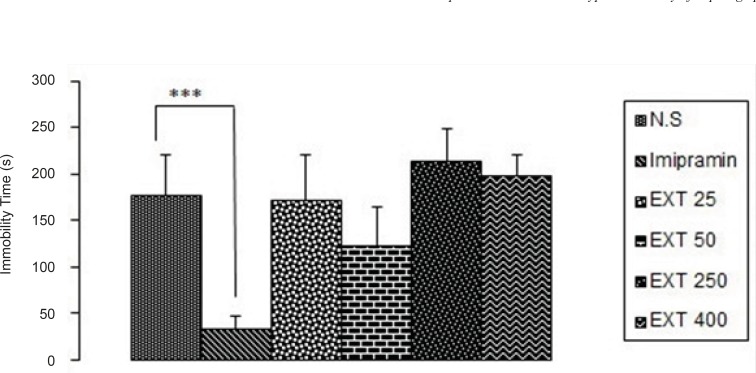
The antidepressant activity of the different doses of *Asperugo procumbents *L. hydroalcoholic extract (25, 50, 250, 400 mg/kg) in forced swimming test. Solvent and different doses of the extract were intraperitoneally administered 45 min before running the test. Measurement was done during 5 min. Imipramine (15 mg/kg) was used as reference drug. Data are mean + SEM. of 5-6 animals in each group. *** p < 0.001 significantly different from control group. EXT: extract

As it is seen in Figure 1, imipramine significantly (p < 0.001) decreased immobility time compared to the control group.None of the doses of the AHE could significantly reduce immobility time in comparison with control group. However, the best result was obtained from the dose 50 mg/kg of the extract.

*Tail suspension test*

**Figure 2:**
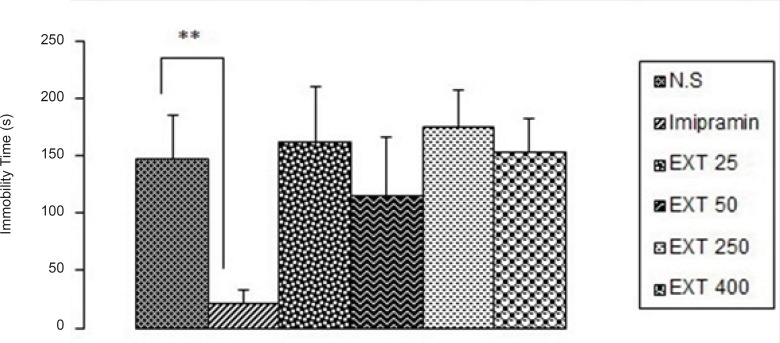
The antidepressant activity of the different doses of *Asperugo procumbensb *L. hydroalcoholic extract (25, 50, 250, 400 mg/kg) in tail suspension test. Solvent and different doses of the extract were intraperitoneally administered 45 min before running the test. Measurement was done during 5 min. Imipramine (15 mg/kg) was used as reference drug. Data are mean + SEM. of 5-6 animals in each group. ** p < 0.01 significantly different from control group. EXT: extract

As it is seen in Figure 2, imipramine significantly (p < 0.01) decreased immobility time compared to the control group, but none of the doses of the AHE could significantly reduce immobility time in comparison with control group. However, the best result was obtained from the dose 50 mg/kg of the extract.

*Pentobarbital-induced hypnotic test*

**Figure 3:**
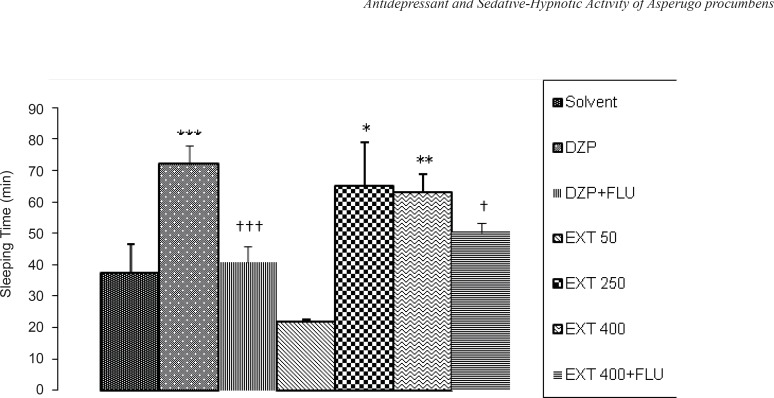
The sedative-hypnotic activity of the different doses of *Asperugo procumbens *L. hydroalcoholic extract (50, 250, 400 mg/kg) in pentobarbital-induced hypnotic test. Solvent, diazepam (2 mg/kg) and different doses of the extract were intraperitoneally administered 30 minutes before challenging animals with IP. injection of pentobarbital (40 mg/kg). Flumazenil (10 mg/Kg) was used 15 min before the extract or diazepam. Data are mean + SEM. of 5-6 animals in each group. * p < 0.05; ** p < 0.01; *** p < 0.001 significantly different from control group.† p < 0.05, ††† p < 0.001 significantly different from the same group plus Flumazenil (10 mg/Kg). DZP: diazepam; EXT: extract; FLU: flumazenil

**Figure 4:**
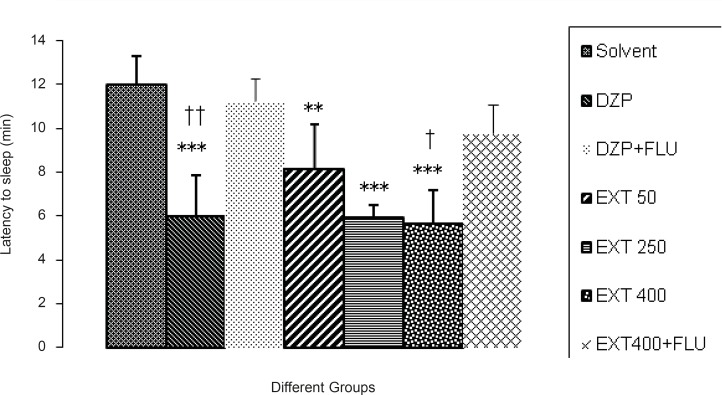
Comparisons between the period of latency to sleep at the different doses of *Asperugo procumbens *L. hydroalcoholic extract (50, 250, 400 mg/kg) in pentobarbital-induced hypnotic test**. **Solvent, diazepam (2 mg/kg) and different doses of the extract were intraperitoneally administered 30 minutes before challenging animals with IP. injection of pentobarbital (40 mg/kg). Flumazenil (10 mg/Kg) was used 15 min before the extract or diazepam. Data are mean + SEM. of 5-6 animals in each group.** p < 0.01, *** p < 0.001 significantly different from control group.† p < 0.05, †† p < 0.01 significantly different from the same group plus Flumazenil (10 mg/Kg). DZP: diazepam; EXT: extract; FLU: flumazenil

The effects of the different doses of AHE on sleeping time and sleep latency induced by pentobarbital are shown in Figure 3 and Figure 4, respectively. AHE at the doses of 250 and 400 mg/kg significantly increased sleeping time compared to the vehicle (p < 0.05 and p < 0.01, respectively) and this effect is nearly equivalent to the reference drug diazepam (2 mg/kg), while AHE at dose 50 mg/kg decreased sleeping time significantly (p < 0.05) compared to the vehicle (not shown on the Figure). In Figure 3 it can be seen that flumazenil (10 mg/kg, IP) reversed the effects of diazepam (p < 0.01) and 400 mg/kg AHE (p < 0.05). So, there was a decreased sleeping time in pentobarbital-induced hypnotic test.

All of the doses of the AHE and diazepam significantly reduced the latency to sleep in comparison to the vehicle.As it is seen in Figure 4, flumazenil (10 mg/kg, IP) reversed the effects of diazepam (p < 0.01) and 400 mg/kg AHE (p < 0.05). So, there was an increased latency to sleepin pentobarbital-induced hypnotic test.

## Discussion

During five thousand years of reco rded history, we know that from the ancient times people have used different methods and procedures in treatment of various psychiatric disorders and very often these were medicinal preparations of plants. Numerous scientific discoveries in the industrial age madea big contribution to medicine development and significantly improved quality of life for psychiatric patients during the last century. However, evidence-based medicine after big bliss faced a lot of disappointments, and an attitude that some natural drugs were unnecessarily thrown out of use step by step came along. On the other hand, there are a huge number of patients that use natural medicinal plants for self-treatment of different psychiatric disorders (17).

Many of today’s synthetic drugs originated from the plant kingdom, and only about two centuries ago, the major pharmacopoeias were dominated by herbal drugs. Herbal medicine went into rapid decline when basic and clinical pharmacology established themselves as leading branches of medicine. Nevertheless, herbal medicine is still of interest in many diseases in particular psychiatric and neurological disorders. Although a multitude of pharmaceutical agents are available for the treatment of mental disorders, physicians find that many patients cannot tolerate the side effects, do not respond adequately, or eventually lose their response. In comparison, many therapeutic herbs have far fewer side effects. They can provide an alternative treatment or be used to enhance the effect of prescription medications (18).

In the present study, we studied antidepressant and sedative-hypnotic activity of the hydroalcoholic extract of *Asperugo procumbens*, in mice. We used two animal models, the forced swimming test (FST) and tail suspension test (TST) for antidepressant study. The immobility displayed by rodents when subjected to unavoidable stress is thought to reflect a state of despair or lowered mood, which are thought to reflect depressive disorders in humans. In addition, the immobility time has been shown to be reduced by treatment with antidepressant drugs. Moreover, a significant correlation was found between the clinical efficacy of antidepressant drugs and their potency in both models (13, 14). In both models, results indicated that none of the doses of the extract could reduce the immobility time significantly compared with the control.

One of the possible reasons for these results is the solvent used for the extraction. Hydroalcoholic solutions are used widely for the extraction. They have the effects of water and alcoholic solutions, prevent from microbial pollution and sedimentation of the goal substance during station. In the present study, we used ethanol 70º as our solvent for extraction that can solve flavonoids. The antidepressant effect of many plants is contributed to their flavonoids content.Whether in this plant antidepressant activity is due to its flavonoids or other component such as essences is not clear and needs to be elucidated. Furthermore, in the present study, the experiments were performed acutely and the route of administration of the extracts was IP which was different from its traditional route in human. Since the route and duration of administration may affect the pharmacokinetics of the active components, the therapeutic doses and the extent of effects of preparations obtained here cannot be extrapolated to human and oral and chronic administration of the extract may have better results (19).

In the second set of experiments, the sedative-hypnotic activity of AHE was evaluated.

Diazepam which belongs to the benzodiazepine group is a central nervous system depressant used in the management of sleep disorders such as insomnia. Benzodiazepines have a binding site on GABA recep tortype-ionophore complex (GABA_A_) (20-22). They decrease activity, moderate excitement and calm the recipient.Substances like diazepam (the reference drug used in this study) reduce onset of and increase duration of barbiturate-induced sleep and reduce exploratory activity possessing potentials as sedative (22). AHE after intraperitoneal administration of 250 and 400 mg/kg doses produced sedative effect similar to that observed with 2 mg/kg diazepam. Diazepam is a very well-known anxiolytic benzodiazepine which produces not only anxiolytic-like effect, but also important sedative action. It is possible that the tranquillizing activity of AHE is mediated by GABAergic system, since it can produce profound sedation in mice (22, 23). The inhibitory action of GABA consists in the opening of chloride channels to allow hyperpolarization of the membrane, leading to CNS depression and resulting in sedative and hypnotic activity. Glutamate and GABA are quantitatively the most important excitatory and inhibitory neurotransmitters, respectively, in the mammalian brain (22). Thus, receptors for these two neurotransmitters are regarded as the important targets for psychotropic drugs. In the test of pentobarbital-induced sleeping in mice, the potentiated effect of AHE in micewas represented. It not only prolonged the sleeping time, but also decreased the latency of falling asleep and increased the sleep onset. Since the effect of barbiturates on the CNS involves activating of the inhibitory GABA ergic system, the result of the present study suggests that some ingredients in AHE produce facilitation of this inhibitory system.

In order to determine if benzodiazepine receptor participates in the hypnotic effects of AHE, flumazenil, a specific antagonist of the benzodiazepine site in the GABA_A_-benzodiazepine receptor complex was administered. Pre-treatment with flumazenil significantly inhibited the effects of AHE on pentobarbital-induced sleeping behavior. So, it is possible that the hypnotic activity of AHE involved in the activation of GABA_A_-benzodiazepine receptors.

Plant compounds such as flavonoids, terpenes and saponins have been found to have hypnotic effect (24, 25). Flavonoids with anxiolytic activities have been described in numerous plant species used in folk medicine to depress the CNS. This effect has been ascribed to their affinity for the central benzodiazepine receptors (24). It could be suggested that flavonoids of the *Asperugo procumbens *contribute to the hypnotic effect of this plant through central benzodiazepine receptors.

In conclusion, this work shows that the hydroalcoholic extract of *Asperugo procumbens *L. has low antidepressant activity in acute animal models of depression, but significant sedative-hypnotic effect. Further chemical and pharmacological analysis of the extract will be performed to isolate and characterize the active components responsible for the sedative and hypnotic effect.

## References

[1] Licinio J, Wong M (1999). The role of inflammatory mediators in the biology of major depression: central nervous system cytokines modulate the biological substrate of depressive symptoms, regulate stressresponsive systems, and contribute to neurotoxicity and neuroprotection. Mol. Psychiatry.

[2] Moallem SA, Hosseinzadeh H, Ghoncheh H (2007). Evaluation of antidepressant effects of aerial parts of Echiumvulgare on mice. Iran. J. Bas. Med. Sci.

[3] Dhingra D, Sharma A (2006). A review on antidepressant plants. Nat. Prod. Radiance.

[4] Freitas AE, Budni J, Lobato KR, Binfaré RW, Machado DG, Jacinto J, Veronezi PO, Pizzolatti MG, Rodrigues AL (2010). Antidepressant like action of the ethanolic extract from Tabebuia avellanedae in mice: Evidence for the involvement of the monoaminergic system. Prog. Neuro-Psychopharmacol. Biol. Psychiatry.

[5] Thomas R, Christopher D (2004). Evolution of insomnia: current status and future direction. Sleep Med.

[6] Phillipson JD (2001). Phytochemistry and medicinal plants. Phytochemistry.

[7] Carlini EA (2003). Plants and the central nervous system. Pharmacol. Biochem. Behav.

[8] Zhang Z (2004). Therapeutic effects of herbal extracts and constituents in animalmodels of psychiatric disorders. Life Sci.

[9] Ahanjan M, Mohana DC, Raveesha KA (2008). Antibacterial activity of Asperugo procumbens L.against some human pathogenic bacteria. African J. Microbiol. Res.

[10] Bagheri M, Regan MS (1994). Study the status and use of medicinal plants in Iran and in the world. Forest. Range. Land. J.

[11] National Research Council, Committee for the Update of the Guide for the Care and Use of Laboratory Animals, National Research Council (2010). Guide for the Care and use of Laboratory animals.

[12] Porsolt RD, Le Pichon M, Jalfre M (1977). Depression: a new animal model sensitiveto antidepressant treatments. Nature.

[13] Porsolt RD, Bertin A, Jalfre M (1977). Behavioural despair in mice: a primary screening test for antidepressants. Acrh.Inter. Pharmacodyn. Ther.

[14] Steru L, Chermat R, Thierry B, Simon P (1985). The tail suspension test: a new method for screening antidepressants in mice. Psychopharmacology.

[15] Machado DG, Kaster MP, Binfaré RW, Dias M, Santos AR, Pizzolatti MG, Brighente IM, Rodrigues A L (2007). Antidepressant-like effect of the extract from leaves of Schinusmolle L. in mice: evidence for the involvement of the monoaminergic system. Prog. Neuropsychopharmacol. Biol. Psychiatry.

[16] Blanco MM, Costa CARA, Freire AO, Santos Jr JG, CostaM (2009). Neurobehavioral effect of essential oil of Cymbopogon citratus in mice. Phytomedicine.

[17] Babić D (2007). Herbal medicine in the treatment of mental disorders. Psychiatr. Danub.

[18] Akhondzadeh S, Maleki J (2006). Herbal medicines in the treatment of psychiatric and neurological disorders. Iran. J. Psychiatry.

[19] Emamghoreishi M, Heidari-Hamedani G (2006). Sedative-Hypnotic Activity of Extracts and Essential Oil of Coriander Seeds. Iran. J. Med. Sci.

[20] Huang F, Xiong Y, Xu L, Ma S, Dou C (2007). Sedative and hypnotic activities of the ethanol fraction from Fructus schisandrae in mice and rats. J. Ethnopharmacol.

[21] Herrera-Ruiz M, Gutiérrez C, Enrique Jiménez-Ferrer J, TortorielloJ, Mirón G, León I (2007). Central nervous system depressant activity of an ethyl acetate extract from Ipomoeastansroots. J. Ethnopharmacol.

[22] Alnamer R, Alaoui K, Bouidida EH, Benjouad A, Cherrah Y (2012). Sedative and hypnotic activities of the methanolic and aqueous extracts of Lavandula officinalis from Morocco. Adv. Pharmacol. Sci.

[23] Gottesmann C (2002). GABA mechanisms and sleep. Neuroscience.

[24] Rakhshandah H, Hosseini M, Dolati K (2004). Hypnotic Effect of Rosa damascena in Mice. Iran. J. Pharm. Res.

[25] Rakotonirina VS, Bum EN, Rakotonirina A, Bopelet M (2001). Sedative properties of the decoction of the rhizome of Cyperus articulatus. Fitoterapia.

